# Comparison of Proangiogenic Effects of Adipose-Derived Stem Cells and Foreskin Fibroblast Exosomes on Artificial Dermis Prefabricated Flaps

**DOI:** 10.1155/2020/5293850

**Published:** 2020-01-31

**Authors:** Jiachao Xiong, Zhixiao Liu, Minliang Wu, Mengyan Sun, Yu Xia, Yuchong Wang

**Affiliations:** ^1^Department of Plastic Surgery, Changhai Hospital, Naval Military Medical University, Shanghai 200433, China; ^2^Department of Histology and Embryology, Naval Military Medical University, Shanghai 200433, China

## Abstract

Large prefabricated flaps often suffer from necrosis or poor healing due to a lack of new blood vessels and related factors that promote angiogenesis. The innovative use of adipose-derived stem cell exosomes (ADSC-Exo) resolves the problem of vascularization of prefabricated flaps. We analyzed the differential microRNA (miRNA) expression in ADSC-Exo using next-generation sequencing (NGS) technology to explore their potential mechanisms in promoting vascularization. We observed that ADSC-Exo could significantly promote the vascularization of artificial dermis prefabricated flaps compared with human foreskin fibroblast exosomes. NGS indicated that there were some differentially expressed miRNAs in both exosomes. Bioinformatics analysis suggested that significantly upregulated hsa-miR-760 and significantly downregulated hsa-miR-423-3p in ADSC-Exo could regulate the expression of the *ITGA5* and *HDAC5* genes, respectively, to promote the vascularization of skin flaps. In summary, ADSC-Exo can promote skin-flap vascularization, and thereby resolve the problem of insufficient neovascularization of artificial dermis prefabricated flaps, thus expanding the application of prefabricated skin-flap transplantation.

## 1. Introduction

Wounds involving large areas of skin and soft tissue caused by trauma, tumor resection, or chronic diseases for various reasons are often difficult to heal, resulting in refractory wounds. Conventional skin transplantation may not be successful for such refractory wounds due to the lack of vascular structure and the inability to reconstruct a blood supply, thus necessitating the use of skin flaps for repair. Although flap transplantation is currently widely used in clinical wound repair [1], the thickness of conventional flaps is limited by the location of the specimen. Moreover, the thickness of the flap is particularly critical for wounds in deep areas, joints, and areas with high wear and weight bearing. Prefabricated flaps thus offer a good method for optimizing traditional flaps.

Prefabricated flaps involve reconstructing an arbitrary skin flap into an axial flap for later wound repair by transplanting known vascular tissue [2]. This technology can increase the selection of skin flaps, allow the accurate design and manufacture of flap size and thickness, and reduce loss and waste of donor tissue. Moreover, it also improves aesthetic and local functional recovery of the tissue after repair and protects the patient from pain associated with a forced position [3]. However, the main problem with prefabricated flaps is currently the limited range of options. Furthermore, large prefabricated flaps often suffer from necrosis or poor healing due to a lack of new blood vessels and related factors that promote angiogenesis.

Adipose-derived stem cells (ADSCs) are stem cells with multidirectional differentiation potential, first isolated by Zuk et al. in 2001 [4]. ADSCs play a definite role in promoting vascularization during tissue repair and reconstruction; however, the mechanism by which they achieve this is unclear. Most researchers currently believe that ADSCs differentiate mainly into vascular endothelial cells and smooth muscle cells to form a new vascular network [5], or secrete paracrine factors, such as basic fibroblast growth factor, vascular endothelial growth factor, hepatocyte growth factor, platelet-derived growth factor, and other angiogenesis-related cytokines and growth factors to promote local microvascularization [6, 7]. ADSC transplantation has achieved better therapeutic effects than current conventional treatment methods in patients with refractory wounds [8]. However, despite the many advantages of ADSCs, technical problems and the risk of tumor formation currently limit their clinical application [9]. Exosomes are membranous vesicles about 30–150 nm in diameter that are released from the intracellular matrix into the extracellular matrix [10]. They can carry a variety of biological macromolecules, including proteins, lipids, and nucleic acids, and participate in various physiological processes, such as the immune response, antigen presentation, and protein and RNA transport [11]. Previous studies reported that interleukin-6 in ADSC exosomes (ADSC-Exo) protected flaps from ischemia-reperfusion injury [12]. However, no studies have reported on the ability of ADSC-Exo to promote angiogenesis in prefabricated flaps.

We therefore applied ADSC-Exo and human foreskin fibroblast exosomes (HFF-Exo) to artificial dermal prefabricated flaps and compared their proangiogenic effects. We also performed next-generation sequencing (NGS) of both types of exosomes and compared the highly enriched microRNAs (miRNAs) and identified differentially expressed miRNAs by quantitative methods. We analyzed the distribution of the target genes using the Gene Ontology (GO) and Kyoto Encyclopedia of Genes and Genomes (KEGG) pathway databases, which indicated that the differentially expressed miRNAs may play an important role in the regulation of gene function.

## 2. Materials and Methods

### 2.1. Isolation and Culture of hADSCs and HFFs

Human subcutaneous adipose tissue and human foreskin tissue samples were obtained from Changhai Hospital affiliated to the Naval Military Medicine University, Shanghai, China. All tissues were sourced after obtaining informed consent from the patients. Primary human ADSCs (hADSCs) and HFFs were generated as described previously [13, 14]. hADSC and HFF pellets were resuspended separately in low-glucose and high-glucose Dulbecco's Modified Eagle's Medium (DMEM) (HyClone, UT, USA) with 2.5% exosome-depleted fetal bovine serum (FBS) (Gibco, Grand Island, NY, USA), and cultured in a humidified incubator containing 5% CO_2_. The medium was changed every 2–3 days after cell attachment.

### 2.2. Exosome Isolation

Exosomes were isolated from hADSCs and HFFs by differential ultracentrifugation, as described previously [15]. In brief, cell culture medium was derived from 80% to 90% confluent hADSCs or HFFs under sterile conditions. Differential ultracentrifugation was performed at 300 × *g* and 2000 × *g* for 10 min to remove dead cells, followed by 10,000 × *g* for 30 min to remove cell debris. The cell pellets were then centrifuged twice for 70 min at 100,000 × *g*. All centrifugations were carried out at 4°C. The pellets were finally resuspended in 100 *μ*l of cold phosphate-buffered saline (PBS) and stored immediately at -80°C and used within 1–2 weeks.

### 2.3. Exosome Identification

#### 2.3.1. Transmission Electron Microscopy (TEM)

The pellets rich in exosomes were diluted in 30 *μ*l of PBS (HyClone, UT, USA) and kept at 4°C until transmission electron microscopy (TEM) analysis. One drop of exosome sample was placed on a carbon-coated copper grid for 5 min and stained with a drop of 2% phosphotungstic acid for 3 min. Excess liquid was removed with absorbent paper, and the sample was air-dried for 15 min. The preparation was then examined by TEM.

#### 2.3.2. Nanoparticle Tracking Analysis (NTA)

NTA was performed using a NanoSight instrument (Zetaview, Particle Metrix, Germany), according to the manufacturer's protocols. The particle size distribution and concentration of all types of nanoparticles with diameters of 10–2000 nm could be analyzed rapidly and automatically by NTA. NTA detection technology also ensured the accuracy and repeatability of the sample readings.

#### 2.3.3. Western Blotting

For western blotting, proteins from each sample were separated by 5% sodium dodecyl sulfate-polyacrylamide gel electrophoresis and transferred to a polyvinylidene difluoride membrane (Cell Signaling Technology, MA, USA) at 300 mA for 100–120 min. The membrane was initially blocked in 5% bovine serum albumin for 2 h at room temperature and then incubated overnight at 4°C with primary heat shock protein- (HSP-) 70 (1 : 2000, Abcam, UK), glyceraldehyde 3-phosphate dehydrogenase (GAPDH) (1 : 1000, Cell Signaling Technology, MA, USA), histone deacetylase 5 (HDAC5) (1 : 1000, CST, USA), or integrin alpha-5 (ITGA5) antibody (1 : 1000, Cell Signaling Technology, MA, USA), respectively, followed by three or four washes with Tris-buffered saline, 0.1% Tween 20. The membrane was then incubated with goat anti-rabbit horseradish peroxidase-conjugated secondary antibody (1 : 3000, Beyotime, China) for 2 h at room temperature. Protein expression was then observed by chemiluminescence using an Alpha Imager scanner (Tecan, Thermo Fisher Scientific, USA).

### 2.4. *In Vivo* Studies

A total of 48 male Sprague-Dawley rats were divided randomly into four groups: an ADSC group, an ADSC-Exo group, an HFF group, and an HFF-Exo group. All the experiments were approved according to the guidelines of the Health Sciences Animal Policy and Welfare Committee of Changhai Hospital affiliated with the Navy Military Medical University.

Artificial dermis prefabricated flap and leg wound rat models were constructed as reported previously [16]. After the graft flap and the abdominal wall wound were sutured, the base of the flaps was multipoint injected with 100 *μ*l of PBS containing 1 × 10^6^ ADSCs or HFFs and 200 *μ*g of ADSC-Exo or HFF-Exo, respectively.

The flaps in the four groups were observed at 7, 14, 21, and 28 days postoperatively. The survival status of the skin flaps was assessed by monitoring surface color, blood supply, and circumference of the incision.

### 2.5. Immunohistochemistry

Flap tissues were excised at 28 days after surgery and analyzed by histological staining. The excised skin flaps were fixed with 10% formalin and dehydrated in graded ethanol. Paraffin-embedded specimens were then cut into 5 *μ*m sections and stained with hematoxylin and eosin and Masson's trichrome for histological observation and to evaluate collagen maturation. Skin-flap angiogenesis was observed by CD31^+^ immunohistochemical staining (1 : 50, Abcam, UK). Tissue-section preparation and immunohistochemical assays were carried out as reported previously [17]. CD31 positivity was indicated by a brown reaction. Photomicrographs were obtained under an optical microscope (Leica, Germany). Four random areas in each section were selected and analyzed using Image-Pro Plus 6.

### 2.6. Flap Microangiography

Postmortem microangiography was performed 7 days after flap transplantation. Rats were injected with 30% barium sulfate solution into the right jugular vein, at low pressure. The flaps were harvested the next day and examined by radiography to reveal the vascular network. Flap microangiography was carried out as reported previously [16].

### 2.7. Effects of ADSC-Exo and HFF-Exo on Proliferation of Human Umbilical Vein Endothelial Cells (HUVECs) *In Vitro*

HUVECs were obtained from the American Type Culture Collection, seeded at 1 × 10^5^ cells per well in 24-well plates, and cultured in high-glucose DMEM with 2.5% exosome-depleted FBS to reach 70%–80% confluency. The cells were then divided into two groups: an ADSC-Exo group and an HFF-Exo group. HUVECs were cocultured with 100 *μ*g/ml ADSC-Exo or HFF-Exo for 24 h, and the effects of the respective exosomes on cell proliferation were detected using a 5-ethynyl-2 deoxyuridine (EdU) assay kit (RiboBio, Guangzhou, China) [18], according to the manufacturer's instructions. The cells were finally observed under a fluorescence microscope (Zeiss HLA100, Shanghai, China). Proliferating HUVECs were indicated by green fluorescence and nuclei by blue fluorescence, and the ratio of these was calculated to obtain the proliferation rate.

### 2.8. Preparation of Libraries and Sequencing

Total exosomal RNA was extracted using TruSeq Small RNA Sample Prep Kits (Illumina, China), according to the manufacturer's instructions. The quality of the RNA was detected by 1% agarose gel electrophoresis and analyzed using an Agilent 2100 Bioanalyzer. The libraries were sequenced using an Illumina HiSeq X Ten platform. Small RNA sequencing and analysis were initially conducted by OE Biotech Co., Ltd. (Shanghai, China). Basic reads were converted into sequence data (raw data/reads) by base calling. Low-quality reads were filtered, and reads with 5′ prime contaminants and poly(A) were removed. Reads without 3′ adapters and insert tags and reads with <15 or >41 nucleotides were filtered, and clean reads were obtained.

### 2.9. Bioinformatics Analysis

The length distributions of the clean sequences in the reference genome were determined. Noncoding RNAs were annotated as rRNAs, tRNAs, small nuclear RNAs (snRNAs), and small nucleolar RNAs. These RNAs were aligned and then subjected to BLAST [19] search against the Rfam v.10.1 (http://www.sanger.ac.uk/software/Rfam) [20] and GenBank databases (http://www.ncbi.nlm.nih.gov/genbank/). Known miRNAs were identified by alignment against the miRBase v.21 database (http://www.mirbase.org/) [21], and the expression patterns of the known miRNAs in different samples were analyzed. Unannotated small RNAs were analyzed by miRDeep2 [22] to predict novel miRNAs. Based on the hairpin structure of a pre-miRNA and the miRBase database, the corresponding miRNA star sequence was also identified. Differentially expressed miRNAs were identified with a threshold *P* value < 0.05. The *P* value was calculated using the DEG algorithm in the *R* package.

### 2.10. Prediction and Functional Analysis of miRNA Target Genes

Target genes of differentially expressed miRNAs were predicted using miRanda software [23] in animals, with the following parameters: S ≥ 150ΔG≤−30 kcal/mol and demand strict 5′ seed pairing. GO enrichment and KEGG pathway enrichment analyses of differentially expressed miRNA target genes were performed using *R*, based on the hypergeometric distribution.

### 2.11. Real-Time Polymerase Chain Reaction (PCR)

Total RNA was isolated from HUVECs using Trizol® Reagent (Life Technologies, USA), according to the manufacturer's instructions. Two micrograms of total RNA was reverse transcribed into cDNA using miRNA-specific primers (hsa-miR-423-3p: MIMAT0001340; hsa-miR-760: MIMAT0004957) with a TaqMan miRNA reverse transcription kit (Thermo Fisher Scientific, USA) [24]. Real-time PCR was then performed with an initial denaturation step at 95°C for 10 min, followed by 40 cycles of 95°C for 15 s and 60°C for 1 min. The level of the U6 small nuclear RNA gene was used as an internal control, and normalized relative expression levels were calculated using the 2^−*∆∆*Ct^ method.

### 2.12. Statistical Analysis

Data were analyzed using SPSS 17.0 and presented as means ± standard deviation. Statistical significance was determined by ANOVA or Student's *t*-test, and the gene expression data set was also analyzed using Spearman's rank test. A value of *P* < 0.05 was considered significant.

## 3. Results

### 3.1. Characterization of Exosomes

**Figure 1:**
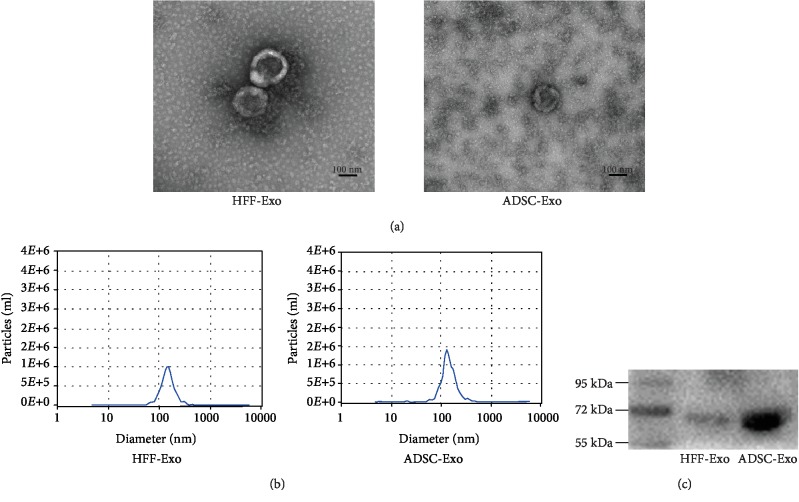
Verification of ADSC-Exo and HFF-Exo. (a) The TEM image of exosomes. (b) Exosome size distribution analysis by NTA. (c) Western blot of exosomes for HSP70.

Exosomes are intracellular vesicles with a diameter of 30–150 nm, which can be obtained from cell culture media by various methods, including ultracentrifugation, ultrafiltration, immunoaffinity capture-based techniques, and microfluidics-based isolation techniques. The exosomes isolated in the current study were verified by TEM, NTA, and western blot. TEM (Figure 1(a)) confirmed the typical morphology of the exosomes, and NTA (Figure 1(b)) showed that the average diameter of ADSC-Exo was 133.6 ± 1 nm and HFF-Exo was 142 ± 5.1 nm.

In addition, western blot (Figure 1(c)) showed that the exosomal marker HSP70 was highly expressed. These results were all consistent with the characteristics of exosomes.

### 3.2. *In Vivo* Studies

**Figure 2:**
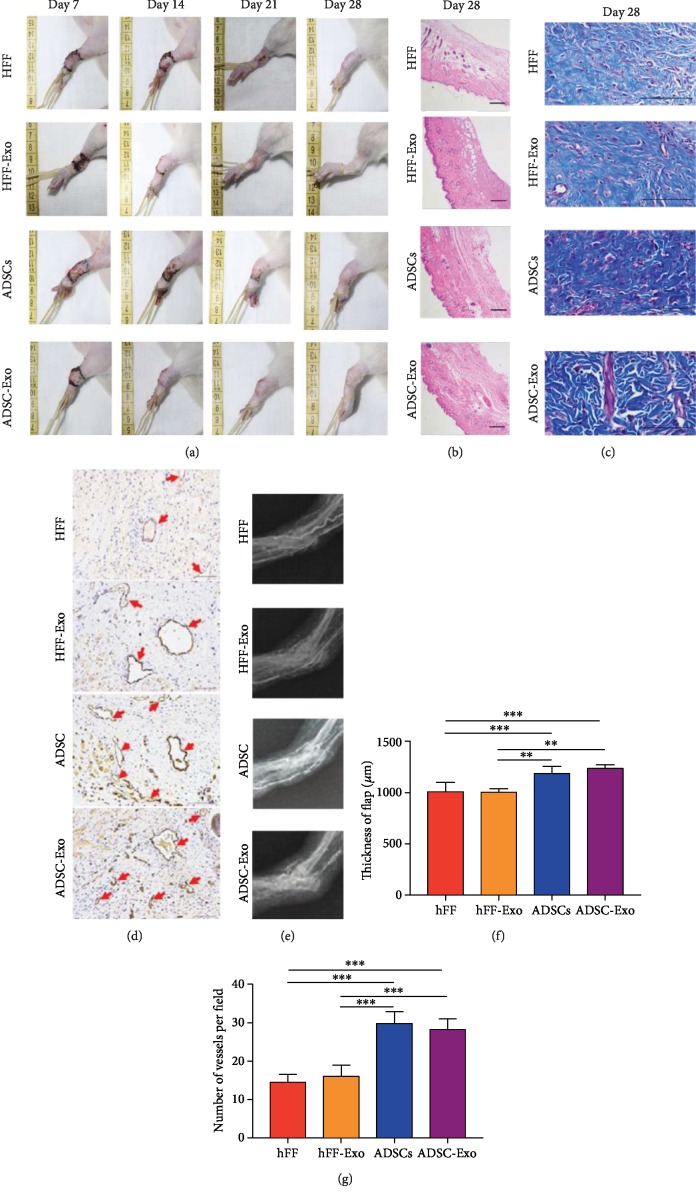
ADSC-Exo promotes repair and vascularization of prefabricated flap graft wounds in vivo. (a) The repair process of prefabricated skin flaps in rats of each group. (b) Microscopic observation of the section showing the flap thickness in four groups. Scale bare = 200 *μ*m. (c) Masson staining for mature collagen. Scale bare = 100 *μ*m. (d) Microscopic observation of CD31 immunostaining. Brown reaction products means CD31-positive immunohistochemical staining and red arrow indicates mature blood vessels. Scale bare = 100 *μ*m. (e) The microvascular angiography of each group. (f) Quantitative analysis of the flap thickness in each group. (g) Quantitative analysis of the number of mature blood vessels. ^∗∗^*P* < 0.01 and ^∗∗∗^*P* < 0.001.

The skin flaps and the epidermal blood supplies in all four groups remained good at the observed time points, with no obvious ulceration or infection. Hair growth was observed on all flaps 21–28 days after the operation (Figure 2(a)).

Microscopic observation of flap sections showed no significant differences in flap thickness and collagen between the ADSC and ADSC-Exo groups, but both of these were significantly better than in the HFF group and HFF-Exo group (Figures 2(b), 2(c) and 2(f)). CD31^+^ immunohistochemical staining (Figures 2(d) and 2(g)) showed that the numbers of blood vessels in the ADSC and ADSC-Exo groups were significantly higher than in the HFF and HFF-Exo groups.

Microvascular angiography performed 28 days after surgery revealed similar degrees of vascularization of the artificial dermis prefabricated flaps in the ADSC-Exo and ADSC groups, and both of these were significantly better than in the HFF and HFF-Exo groups (Figure 2(e)).

### 3.3. NGS of Small RNA Composition in ADSC-Exo and HFF-Exo

**Figure 3:**
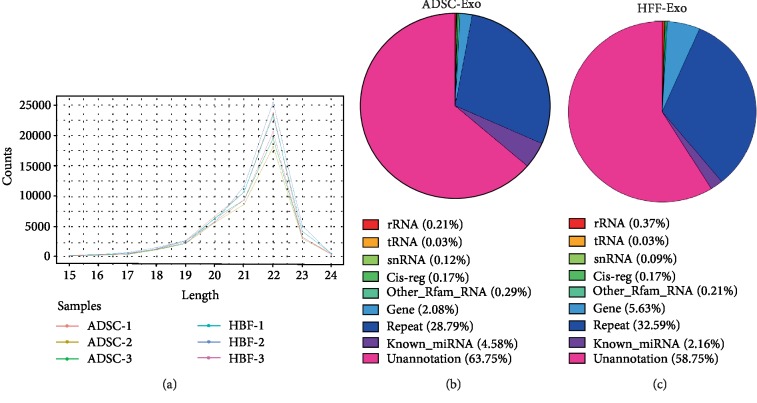
Next-generation sequencing of ADSCs and HFF exosomal miRNA. (a) Line graph of known miRNA lengths in each sample. (b and c) Pie chart of classification annotation of small RNA in ADSC-Exo exosomes and HFF-Exo exosomes.

Small RNAs include miRNAs, tRNAs, rRNAs, piwi-interacting RNAs, snRNAs, and others. miRNAs are noncoding single-stranded RNA molecules approximately 22 nucleotides in length, which are encoded by endogenous genes and are involved in the regulation of posttranscriptional gene expression in plants and animals. To classify the small RNAs in the sequencing results, we compared clean reads using the Rfam database [20], cDNA sequence, species repeat library [25], and miRBase database [21, 26]. The length distribution statistics of the known miRNAs in each sample are shown by a line graph (Figure 3(a)). Furthermore, the distribution of small RNAs in each sample based on the above databases is summarized and displayed as a pie chart (Figures 3(b) and 3(c)).

#### 3.3.1. miRNA Expression Analysis

**Figure 4:**
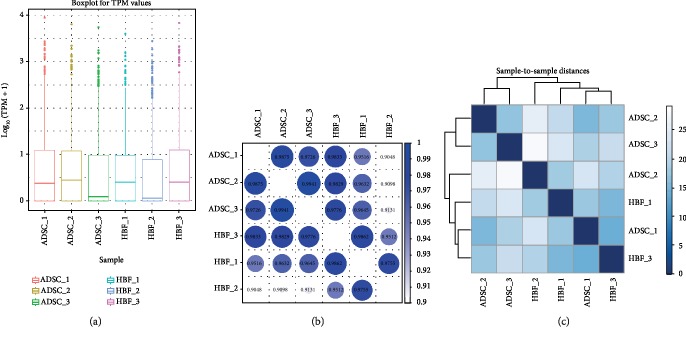
The analysis of miRNA expression. (a) Box line graph of miRNA expression in each sample. (b and c) Correlation coefficient thermogram and sample-to-sample cluster analysis in each sample.

The abundance of a miRNA is directly proportional to its expression level. Small RNA sequencing analysis allows miRNA expression levels to be estimated by locating the mature sequence and counting the newly predicted miRNA sequences. Based on the identified known miRNAs and the newly predicted miRNAs, miRNA expression was calculated as transcript per million [27]. Information on the symmetry and dispersion of the data was displayed by miRNA expression boxplots (Figure 4(a)). When the number of samples was large (≥3), the correlation of miRNA expression levels among samples was an important indicator of the reliability of the experiments and the rationality of sample selection. Similarities between samples were tested using a correlation coefficient thermogram (Figure 4(b)) and sample-to-sample cluster analysis (Figure 4(c)); the closer the sample correlation coefficient was to one or the preferential aggregation, the higher the similarity of expression patterns between the samples.

#### 3.3.2. Differential miRNA Analysis

**Figure 5:**
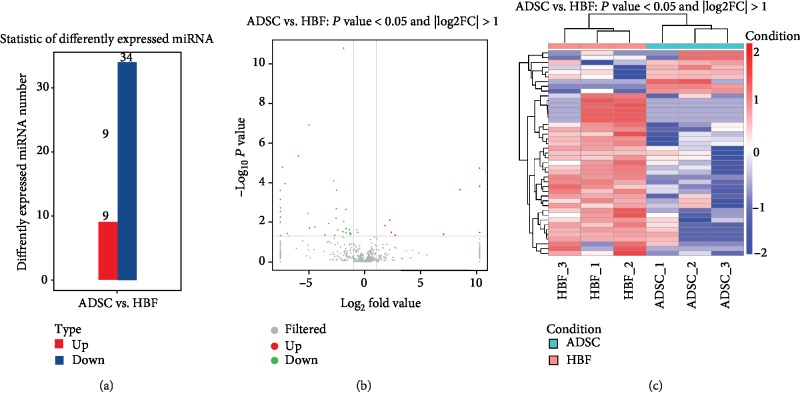
The differential analysis of miRNA. (a) Column graphs show screened differentially expressed miRNA in ADSC-Exo exosomes and HFF-Exo exosomes. (b) Volcanic maps show the overall distribution of differentially expressed miRNAs. Gray represents nondifferentiated miRNA, red represents upregulation of significant differences in miRNA, and green represents downregulation of significant differences in miRNA. (c) Heat map display expression pattern clustering analysis. Red indicates high expression and blue indicates low expression.

Differential expression analysis was used to identify miRNAs that were differentially expressed between different samples. For paired samples with biological replicates, DESeq2 [28] in *R* was used for differential miRNA screening and it revealed a total of 43 differentially expressed genes, including nine upregulated and 34 downregulated genes (Table 1). The differential miRNAs screened between different samples are shown in a histogram (Figure 5(a)), and a volcano plot (Figure 5(b)) was constructed to clarify the overall distribution of the differentially expressed miRNAs. A heat map (Figure 5(c)) was used to show the differential expression of miRNAs according to unsupervised hierarchical clustering. The same types of samples could generally be clustered in the same cluster, and miRNAs in the same cluster may have similar biological functions.

### 3.4. Prediction of Target Genes

Although plant miRNAs are almost perfectly complementary to their target mRNAs, most animal miRNAs are not perfectly complementary. Plant miRNAs can bind to any region of the target mRNA (primarily the protein coding region) and regulate gene expression by cutting the target mRNA or inhibiting its translation. In addition, animal miRNAs have been reported to target the 5′ end of the RNA as well as the coding region. We predicted the miRNA target genes using the miRanda algorithm [29]. The target gene prediction results are shown in Table 2 and in the Supplementary Materials ([Supplementary-material supplementary-material-1]).

### 3.5. Functional miRNA Analysis

**Figure 6:**
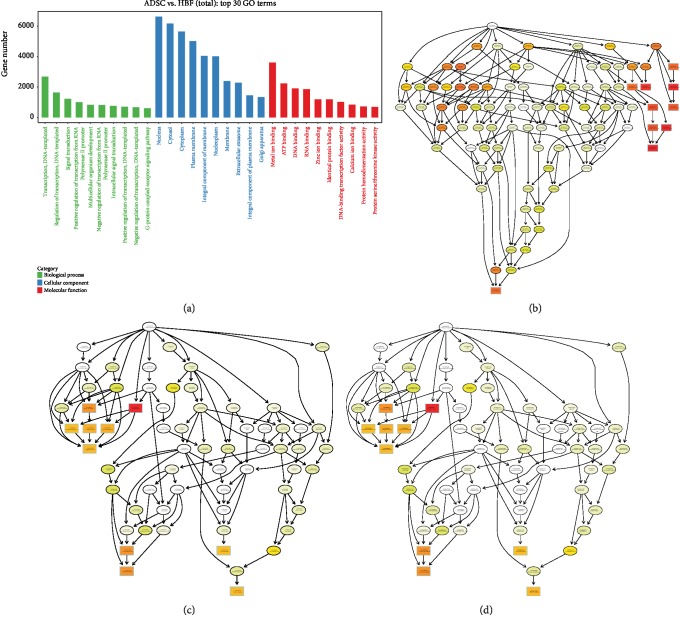
Target gene GO enrichment analysis. (a) Bar chart represents the top 10 GO entries in the three categories. (b–d) Target gene topGO directed acyclic graph. The biological process (a), cellular component, (b) and molecular function (c) of target genes predicted by differential miRNAs in each group. Each GO term is enriched, and the most prominent 10 nodes are represented by a rectangle. The color of the rectangle represents enrichment significance, and the higher the saliency from yellow to red. The basic information of each node is displayed in the corresponding graph, which is GO ID and GO term.

Biological processes, cellular components, and molecular functions were the three most important categories of potential gene functions identified using the GO approach. We screened the top 10 GO entries in the three categories (Figure 6(a)). We also used Fisher's algorithm to perform cell composition, biological process, and molecular function enrichment analyses of the target genes predicted for the differentially expressed miRNAs in each group, and created an acyclic graph using topGO (Figures 6(b)–6(d)). This acyclic graph provides a graphical representation of the target gene GO enrichment analysis results, including the GO nodes and their hierarchical relationships of target gene enrichment. miRNA differential analysis combined with target gene enrichment analysis showed that hsa-miR-760 and hsa-miR-423-3p were associated with the *ITGA5* and *HDAC5* genes, respectively. *ITGA5* is involved in wound repair and vascularization [30], and upregulation of hsa-miR-760 may promote the expression of *ITGA5*, thereby accelerating wound vascularization and healing. In contrast, *HDAC5* has a negative effect on angiogenesis [31], and downregulation of hsa-miR-423-3p may promote wound vascularization by reducing the expression of *HDAC5*.

### 3.6. miRNA Pathway Analysis

**Figure 7:**
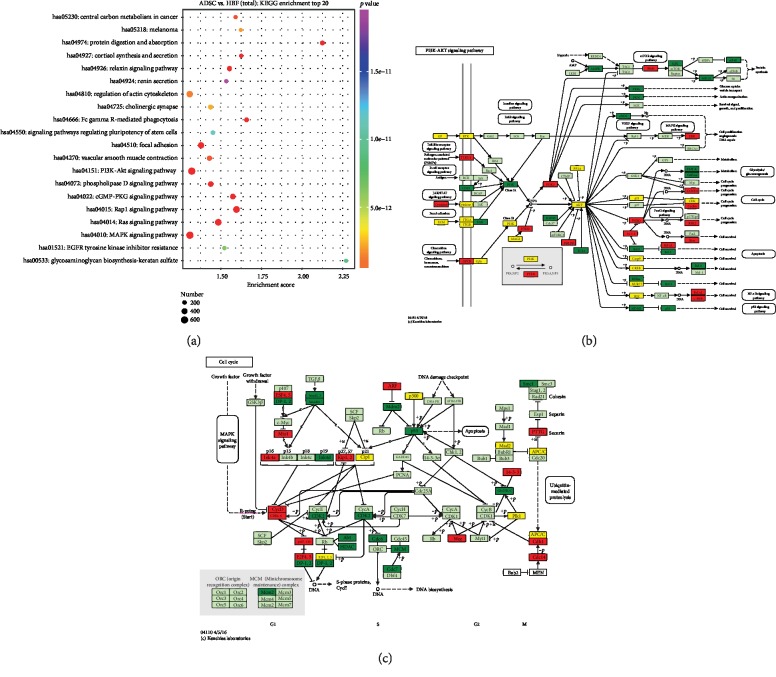
Target gene KEGG enrichment analysis. (a) The top 20 pathway enrichment analysis of differential expression genes by the KEGG database. Each dot in the figure corresponds to a pathway. The order of *P* value from small to large corresponds to red, orange, yellow, green, blue, indigo, and purple, respectively. In brief, the color tends to be red, the smaller the *P* value. In addition, the larger the dot, the more genes in the pathway. (b–c) The PI3K-Akt (b) and MAPK (c) pathway in the KEGG database. Red indicates upregulated genes, green indicates downregulated genes, and yellow indicates both upregulated and downregulated genes.

Pathway analysis helps to identify the cellular pathways involving the differentially expressed miRNAs. We selected the 20 most significant signal pathways (Figure 7(a)), among which phosphatidylinositol-3-kinase-protein kinase B (PI3K-Akt) (Figure 7(b)) and mitogen-activated protein kinase (MAPK) (Figure 7(c)) showed the greatest difference between ADSC-Exo and HFF-Exo in promoting the survival of prefabricated skin flaps. Several studies [32, 33] have reported that the PI3K-Akt and MAPK signaling pathways are closely related to wound vascularization. The differential miRNAs may thus enhance endothelial cell proliferation and motility and promote angiogenesis by activating the PI3K-Akt and MAPK signaling pathways.

### 3.7. ADSC-Exo Regulate miRNA and Gene Expression Affecting HUVEC Proliferation

**Figure 8:**
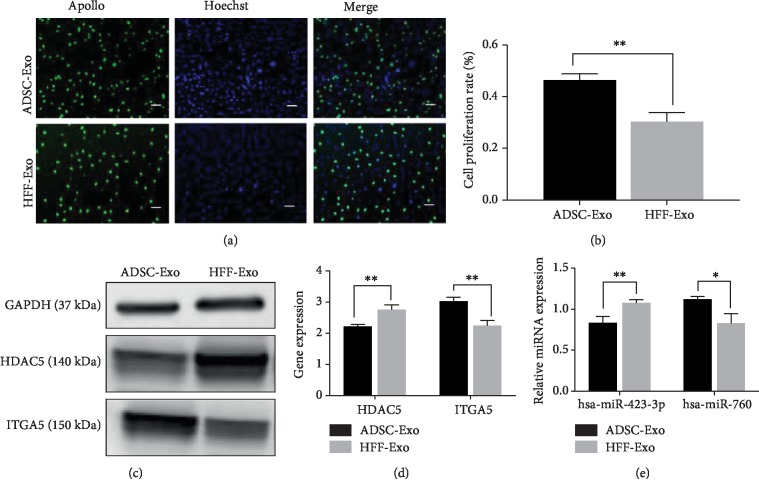
ADSC-Exo promote HUVEC proliferation and regulate expression of related genes. (a) Proliferating HUVECs were stained with green fluorescence, and all nuclei were stained with blue fluorescence. (b) Comparison of cell proliferation rates between the ADSC-Exo group and HFF-Exo. Scale bars indicate 100 *μ*m. (c and d) The expression levels of *ITGA5* and *HDAC5* genes in HUVECs treated with ADSC-Exo or HFF-Exo. (e) The relative miRNA expression levels of hsa-miR-423-3p and hsa-miR-760 in HUVECs treated with ADSC-Exo or HFF-Exo. ^∗^*P* < 0.05 and ^∗∗^*P* < 0.01.

We evaluated the effects of ADSC-Exo and HFF-Exo on HUVEC proliferation by EdU assays. The rate of cell proliferation (Figures 8(a) and 8(b)) was significantly higher in the ADSC-Exo compared with the HFF-Exo group (*P* < 0.01).

We also investigated the effects of *ITGA5* and *HDAC5* on vascularization by coculturing HUVECs with 100 *μ*g/ml ADSC-Exo or HFF-Exo for 72 h, extracting total RNA and protein, and detecting the expression levels of the above miRNAs. Expression levels of hsa-miR-423-3p and *HDAC5* were significantly lower in the ADSC-Exo compared with the HFF-Exo group, while expression levels of hsa-miR-760 and *ITGA5* were significantly higher in the ADSC-Exo group (*P* < 0.01) (Figures 8(c)–8(e)). These results suggested that ADSC-Exo promote neovascularization of artificial dermal prefabricated flaps by regulating the expression of *ITGA5* and *HDAC5*.

## 4. Discussion

Prefabricated flaps were first used successfully in animal models in the 1970s and have since been continuously refined for clinical use. Prefabricated flaps expand the range of the flap donor area and provide a more adequate and stable blood supply. However, prefabricated flaps have limited thickness and are not wear resistant, and are thus not suitable for the repair of certain areas such as joints. Artificial dermis can increase tissue thickness making the repaired areas wear resistant, but limiting contracture. New prefabricated flaps involving a combination of artificial dermis and prefabricated flaps can improve blood perfusion, but their application is limited by the lack of factors to promote neovascularization and angiogenesis. Recent studies showed that miRNAs and proteins contained in exosomes acted as mediators of intercellular information transmission [34, 35]. In addition, numerous studies have shown that stem cell exosomes can accelerate wound repair by promoting wound vascularization, with no risk of stem cell-induced tumorigenesis [36, 37]. However, the proangiogenic effects of ADSC-Exo in prefabricated flaps have not previously been reported. Therefore, we constructed artificial dermal prefabricated flaps and transplanted them into wounds in a rat model, and injected them with ADSCs, HFFs, ADSC-Exo, and HFF-Exo. We observed and compared the vascularization of the prefabricated flaps in each group. There was no significant difference in the thickness or vascularization of the artificial dermal prefabricated flaps between the ADSC and ADSC-Exo groups (*P* > 0.05), and both of these performed significantly better than the HFF and HFF-Exo groups (*P* < 0.05). ADSC-Exo thus significantly promoted vascularization and improved survival of the transplanted artificial dermal prefabricated flaps.

Previous studies [16] showed that the thickness and blood perfusion of artificial dermal prefabricated flaps were superior to conventional prefabricated flaps, but the application of artificial dermal prefabricated flaps was limited by the lack of neovascularization and proangiogenic factors. Exosomes are factors/molecules secreted by cells into the extracellular space, which play a role in intercellular communication. They can be extracted from cell culture media by various methods, including centrifugation, filtration, and ion-exchange chromatography [10]. The size of exosomes can be identified by TEM and NTA, and proteins such as CD63, CD81, TSG101, and HSP70, which are usually rich in exosomes, can be identified by western blot [15]. Numerous recent studies [38–40] showed that mesenchymal stem cell-derived exosomes (MSC-Exo) promoted the repair of tissue damage, leading to great progress in wound repair. MSC-Exo were also shown to induce the proliferation and migration of vascular endothelial cells, promote angiogenesis, and reduce apoptosis of endothelial cells, indicating important roles in injury repair and vascularization [10, 39]. In the current study, we therefore applied ADSC-Exo to artificial dermal prefabricated flaps to overcome the issue of insufficient vascularization. Compared with HFF-Exo, the application of ADSC-Exo to the artificial dermal prefabricated flap resulted in better flap repair and vascularization. However, the specific mechanism by which ADSC-Exo promotes the vascularization of artificial dermal prefabricated flaps remains unclear.

The above results suggest that ADSC-Exo may play an important role in the vascularization of prefabricated flaps. We therefore used NGS technology to analyze the spectrum of miRNAs in ADSC-Exo and HFF-Exo, and identified nine upregulated and 34 downregulated miRNAs (inclusion criteria: ∣log2 (fold change)∣ ≥ 1 and *P* < 0.05). We then analyzed the GO enrichment and KEGG pathways of the differentially targeted miRNA genes, and identified hsa-miR-760 and hsa-miR-423-3p as target genes closely related to *ITGA5* and *HDAC5*, respectively. These two genes also play important roles in regulating angiogenesis through various signaling pathways, such as Smad and MAPK. Fibronectin is involved in the regulation of angiogenesis, and fibronectin in the extracellular matrix can induce neovascularization through the activation of endothelial cells by the *ITGA5* gene [41]. In addition, *ITGA5* has been shown to be closely related to transforming growth factor- (TGF-) *β* superfamily signaling. *ITGA5* can increase the activation of the TGF-*β*-induced Smad signaling pathway and promote the formation of new blood vessels [42]. Meanwhile, the formation of new blood vessels is regulated by the proangiogenic factor fibroblast growth factor 2 and the guidance factor slip2. *HDAC5* inhibits endothelial cell angiogenesis gene expression, which may impede the formation of blood vessels by reducing the effects of FGF2 and slip2 [31]. We therefore hypothesized that upregulation of hsa-miR-760 and downregulation of hsa-miR-423-3p in ADSC-Exo may promote the vascularization of prefabricated flaps through *ITGA5* and *HDAC5*. We subsequently cocultured HUVECs with ADSC-Exo or HFF-Exo to determine their effects on cell proliferation, and detected the relative miRNA expression levels of hsa-miR-423-3p and hsa-miR-760 and the gene expression levels of *ITGA5* and *HDAC5*. These results confirmed the claim that ADSC-Exo promote the proliferation of vascular endothelial cells by regulating the expression of *ITGA5* and *HDAC5*, thereby increasing the neovascularization of artificial dermal prefabricated flaps. According to KEGG analysis results, the target genes of the differential miRNAs were mainly enriched in the PI3K-Akt and MAPK pathways, and also in focal adhesion, Ras signaling, and vascular smooth muscle contraction pathways, which are closely related to cell proliferation and migration. Many studies [32, 33] have shown that the activation of these pathways can significantly promote angiogenesis and promote wound repair. We therefore suggest that ADSC-Exo are affected by the ischemic environment, leading to the enrichment and activation of the differential miRNAs in the above pathways, and thus promoting vascularization of the prefabricated flaps. Many other differentially expressed miRNAs in ADSC-Exo are associated with the vascularization of flaps, and further studies are needed to explore the mechanisms of these exosomal miRNAs in the vascularization of prefabricated flaps.

In conclusion, the application of exosome miRNAs may provide a new strategy to support the application of artificial dermal prefabricated flaps.

## Figures and Tables

**Table 1:** Differentially expressed miRNAs in ADSC-Exo and HFF-Exo.

miRNA_ID	Up-down	Log2 (fold change)	*P* value
hsa-miR-144-3p	Up	1.782035743	0.01482335
hsa-miR-323a-5p	Up	Inf	0.000151549
hsa-miR-379-3p	Up	2.700663628	0.045010729
hsa-miR-671-3p	Up	7.027593698	0.040906496
hsa-miR-760	Up	Inf	0.03275833
novel105_mature	Up	8.49513558	0.000229118
novel58_mature>novel59_mature	Up	2.351421613	0.032363393
novel81_mature	Up	2.233321357	0.007874165
novel91_mature	Up	10.25210635	1.88*E*‐05
hsa-miR-1180-3p	Down	-2.70733413	0.04691167
hsa-miR-1285-3p	Down	-Inf	0.001897423
hsa-miR-1296-5p	Down	-Inf	0.000249769
hsa-miR-1301-3p	Down	-4.972567897	0.020496219
hsa-miR-145-5p	Down	-2.542765565	0.000246944
hsa-miR-151a-3p	Down	-2.432074166	0.024160449
hsa-miR-155-5p	Down	-2.571106574	0.00206618
hsa-miR-181a-2-3p	Down	-6.963036424	0.036476532
hsa-miR-193a-5p	Down	-7.396231592	1.70*E*‐05
hsa-miR-21-3p	Down	-5.958067111	4.56*E*‐06
hsa-miR-221-3p	Down	-1.416445391	0.023844747
hsa-miR-222-5p	Down	-5.041521211	1.26*E*‐07
hsa-miR-28-3p	Down	-1.69545148	0.020763826
hsa-miR-320b	Down	-3.247396762	0.011756834
hsa-miR-409-3p	Down	-1.256733189	0.038090088
hsa-miR-423-3p	Down	-1.321533022	0.041698433
hsa-miR-423-5p	Down	-1.895954828	0.009164352
hsa-miR-425-3p	Down	-Inf	0.005656807
hsa-miR-432-5p	Down	-2.593847518	0.006189466
hsa-miR-450b-5p	Down	-Inf	0.009986907
hsa-miR-483-5p	Down	-Inf	0.047965391
hsa-miR-484	Down	-1.702490895	0.034124196
hsa-miR-493-5p	Down	-4.478027604	0.017697307
hsa-miR-656-3p	Down	-7.578421186	0.002905602
hsa-miR-665	Down	-Inf	0.000718759
hsa-miR-889-3p	Down	-Inf	0.005492105
hsa-miR-92b-3p	Down	-1.340141628	0.034863794
hsa-miR-941	Down	-5.1122535	0.003564468
novel104_mature	Down	-1.918135487	1.67*E*‐11
novel21_mature	Down	-1.701858463	0.002261589
novel22_mature	Down	-2.813031267	8.16*E*‐05
novel27_mature	Down	-3.58318806	0.044512343
novel2_mature	Down	-7.154420814	0.000112645
novel75_mature	Down	-1.983003576	0.030260576

**Table 2:** Part of the differential miRNA target gene in ADSC-Exo and HFF-Exo.

miRNA_ID	Transcript	Total score	Total energy	miRNA length
hsa-miR-423-5p	NM_000179.2	168	-32.29	23
hsa-miR-423-5p	NM_000259.3	158	-30.19	23
hsa-miR-423-5p	NM_000278.3	179	-30.04	23
hsa-miR-423-5p	NM_000296.3	168	-30.13	23
hsa-miR-618	NM_001134440.1	184	-31.94	23
hsa-miR-618	NM_001134441.1	184	-31.94	23
hsa-miR-618	NM_001134442.1	184	-31.94	23
hsa-miR-618	NM_001282880.1	184	-31.94	23
hsa-miR-769-5p	NM_000201.2	175	-31.75	22
hsa-miR-769-5p	NM_000435.2	166	-30.43	22
hsa-miR-769-5p	NM_000898.4	173	-31.79	22
hsa-miR-769-5p	NM_001005751.2	175	-32.22	22
hsa-miR-361-3p	XM_011548019.1	157	-30.22	23
hsa-miR-361-3p	XM_011548020.1	157	-30.22	23
hsa-miR-361-3p	XM_011548208.1	157	-30.22	23
hsa-miR-361-3p	XM_011548209.1	157	-30.22	23
hsa-miR-361-3p	XM_011548515.1	164	-31.01	23
hsa-miR-361-3p	XM_011548516.1	164	-31.01	23
hsa-miR-361-3p	XM_011548517.1	164	-31.01	23
hsa-miR-361-3p	XM_011548518.1	164	-31.01	23

## Data Availability

The next generation sequencing data used to support the findings of this study were supplied by Yuchong Wang under license and so cannot be made freely available. Requests for access to these data should be made to [Yuchong Wang, drwangyc@163.com].

## Abbreviations

ADSCs: Adipose-derived stem cells
ADSC-Exo: Adipose-derived stem cell exosomes
bFGF: Basic fibroblast growth factor
CM: Culture media
DMEM: Dulbecco's Modified Eagle's Medium
FBS: Fetal bovine serum
GO: Gene Ontology
HE: Hematoxylin and eosin
HFF: Human foreskin fibroblasts
HFF-Exo: Human foreskin fibroblast exosomes
HGF: Hepatocyte growth factor
HUVEC: Human umbilical vein endothelial cells
IL: Interleukin
KEGG: Kyoto Encyclopedia of Genes and Genomes
MAPK: Mitogen-activated protein kinase
MSC-Exo: Mesenchymal stem cell-derived exosomes
NGS: Next-generation sequencing
NTA: Nanoparticle tracking analysis
PDGF: Platelet-derived growth factor
PI3K-Akt: Phosphatidylinositol-3-kinase-protein kinase B
TEM: Transmission electron microscopy
TGF-*β*: Transforming growth factor-*β*
TPM: Transcript per million
VEGF: Vascular endothelial growth factor.

## Supplementary Materials

Supplementary Materials The supplementary material is the raw data of the target gene prediction result.
